# Case Report: Occupational therapy in a patient with an overgrowth syndrome that restricts movement

**DOI:** 10.12688/f1000research.21348.1

**Published:** 2019-12-03

**Authors:** Luca Coppeta, Sandro Gentili, Francesca Papa, Ludovico Maria De Zordo, Stefano Mugnaini, Antonio Pietroiusti

**Affiliations:** 1 Occupational Medicine, University of Rome Tor Vergata, Rome, Italy, 00133, Italy

**Keywords:** Proteus syndrome, SEMG, overgrowth, ergonomics

## Abstract

**Background: **Overgrowth syndromes are a heterogeneous group of conditions characterized by excessive body growth - localized or generalized - commonly associated with various malformities and an increased oncological risk.

**Case report:** Here we present the case of a 57-year old man, employed in an office, who suffers from an asymmetric overgrowth of the lower limbs. Currently the patient presents malformations of the lower left arm (hip, knee and ankle), evident on the articular and periarticular level, where there are diffuse exostoses. This case discusses the main occupational concerns relating to the patient’s workspace at a high floor level that could create critical issues in the event of an emergency exodus. Given the impossibility of placing the patient in heavy manual activities, employment is limited to office activities. Adjustments were carried out at the patient’s workstation, and thus the patient has been recognized as fit to work. Increased frequency of breaks were prescribed in order to allow the physiological alternation of postures.

**Conclusions: **In cases of overgrowth syndromes, the exact identification of the limitations presented by the patient and observations about ambulatory functions must be carefully evaluated in order to modulate the work environment.

## Introduction

Overgrowth syndromes are a heterogeneous group of conditions characterized by excessive body growth - localized or generalized - commonly associated with various abnormalities and an increased oncological risk ^1^. The classification of this group of disorders has proved to be very complex, both due to the extensive overlap of their clinical characteristics and to the fact that the causative molecular anomalies are not fully known ^2^. Although rapid progress has been made in delineating the causal molecular defects of the most common overgrowth syndromes in the last decade ^1^, there is still much to be done and numerous conditions still need to be carefully understood both phenotypically and genetically.

It is important to underline that most of the overexploited syndromes continue to be diagnosed on the basis of clinical criteria and the genetic identification of typical molecular alterations represent a diagnostic confirmation ^3^. Rapid technological progress currently being made with genetic analysis will probably allow doctors to use more genetic data in the diagnostic approach in the future, and therefore improve the classification of these disorders ^4^.

Overgrowth syndromes are characterized by an increased body size in terms of stature, whether it is generalized or localized, and if there is an increase of the cranial circumference (macrocephaly). To distinguish overgrowth from certain endocrinological (hormonal) disorders, both acquired and genetic, which may induce an increase in growth rate (such as pituitary gigantism, caused by excess growth hormone), the hyperaccumulation syndrome remains by definition non-hormonal. There may also be mental retardation, although cognitive delay is not a constant feature of the syndrome,. as well as dysmorphisms.

The simplest and most practical classification model is based on the type of excessive growth, general and localized, and the part of the body the overgrowth is located on. For example, generalized overgrowth may relate to Beckwith-Wiedemann syndrome, Simpson-Golabi-Behmel, or Perlman, while overgrowths associated with macrocephaly could be Sotos syndrome, and Bannayan-Riley-Ruvalcaba. Hyper-fattening limited to a segment of the body is referred to as isolated hyperplasia.

Here, we report a clinical case regarding the occupational issue and ergonomics adjustments for a patient affected by a rare form of overgrowth syndrome with asymmetric growing of the lower limbs. 

## Case report

### Patient information

Our patient is a man aged 59 years, and is currently employed in an office, who suffers from an asymmetric overgrowth of the lower limbs. This pathology is benign and localized. The abnormal growth process of the left leg began at the age of 18 months and led to the amputation of the distal phalanx of the second finger, which was repeated for recurrence at the age of 4 years. At 35 years the patient underwent subtotal amputation of the first finger and the removal of calcification of the back of the left foot. At the age of 40, he underwent surgical revision of the first finger and amputation of the distal third and fourth finger phalanges.

Over the years, cerebriform connective tissue nevi (CCTNs) has developed on the left upper limb and has undergone surgical treatment of the suspected malignant nevi.

### Clinical findings

The patient’s speech is fluent and his understanding complete. He presents with good personal and social fulfillment (there are no malformations that can disturb the aesthetic appearance of the face). General conditions of the patient are good, from the diagnostic tests performed (medical visit, E.K.G., ultrasonography, spirometry) there are no alterations of the cardio-circulatory system, pulmonary or sensory apparatus. Laboratory tests are normal.

**Figure 1.**
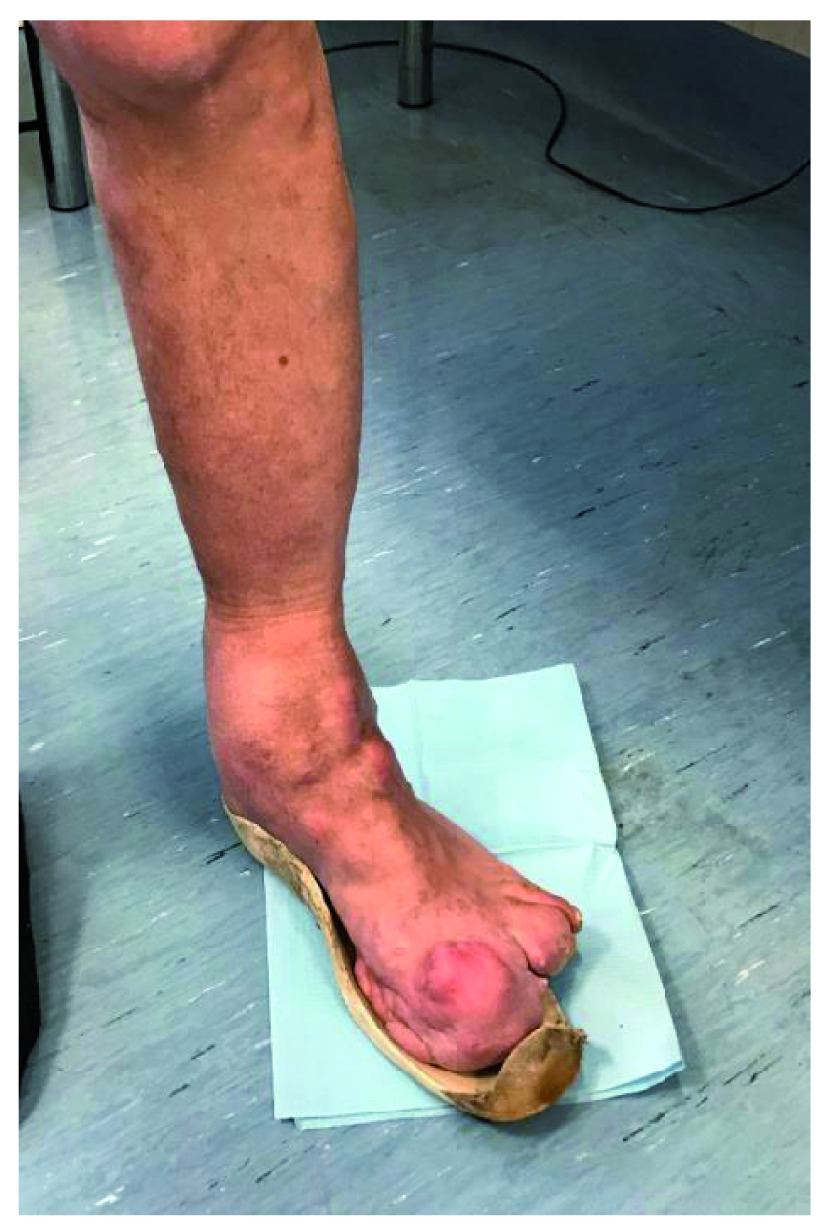
At the level of the medial tibial segment, bone exostosis can be observed on the deep planars, ~4 cm of diameter.

**Figure 2.**
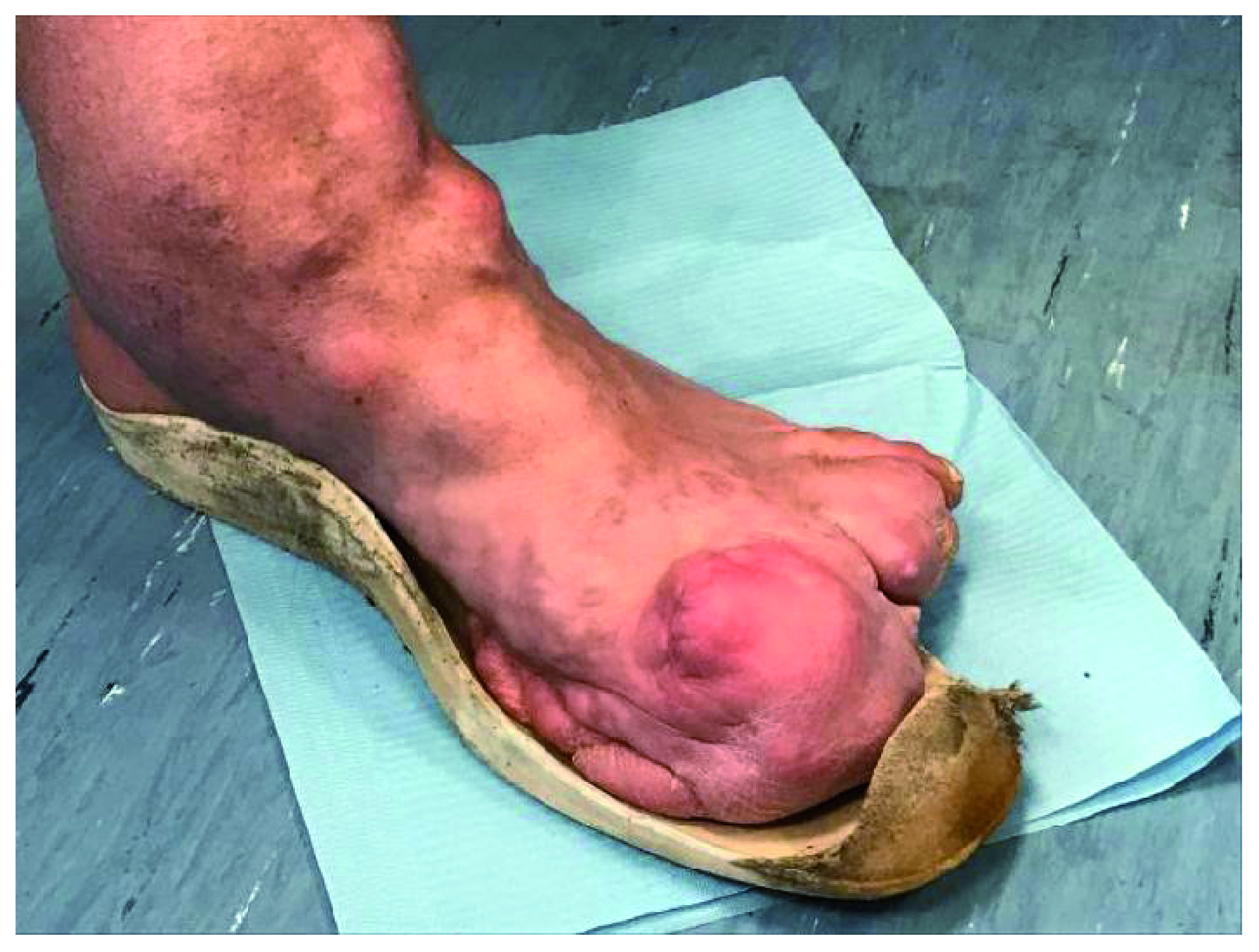
Amputation of the distal phalanges of the first, second, third and fourth fingers, repeated due to recurrence.

Currently the patient presents with malformations of the lower left arms (hip, knee and ankle), evident on the articular and periarticular level, where there are diffuse exostoses. At the hip level, the exostotic formations do not result in reductions of the joint range of motion (ROM). More serious is the situation proceeding distally, where the knee malformations reduce the ROM in a range between 27 and 90 degrees in flexion-extension. At the level of the medial tibial segment a bone exostosis is found on the deep planars, ~4 cm of diameter ( Figure 1, Figure 2).

**Figure 3.**
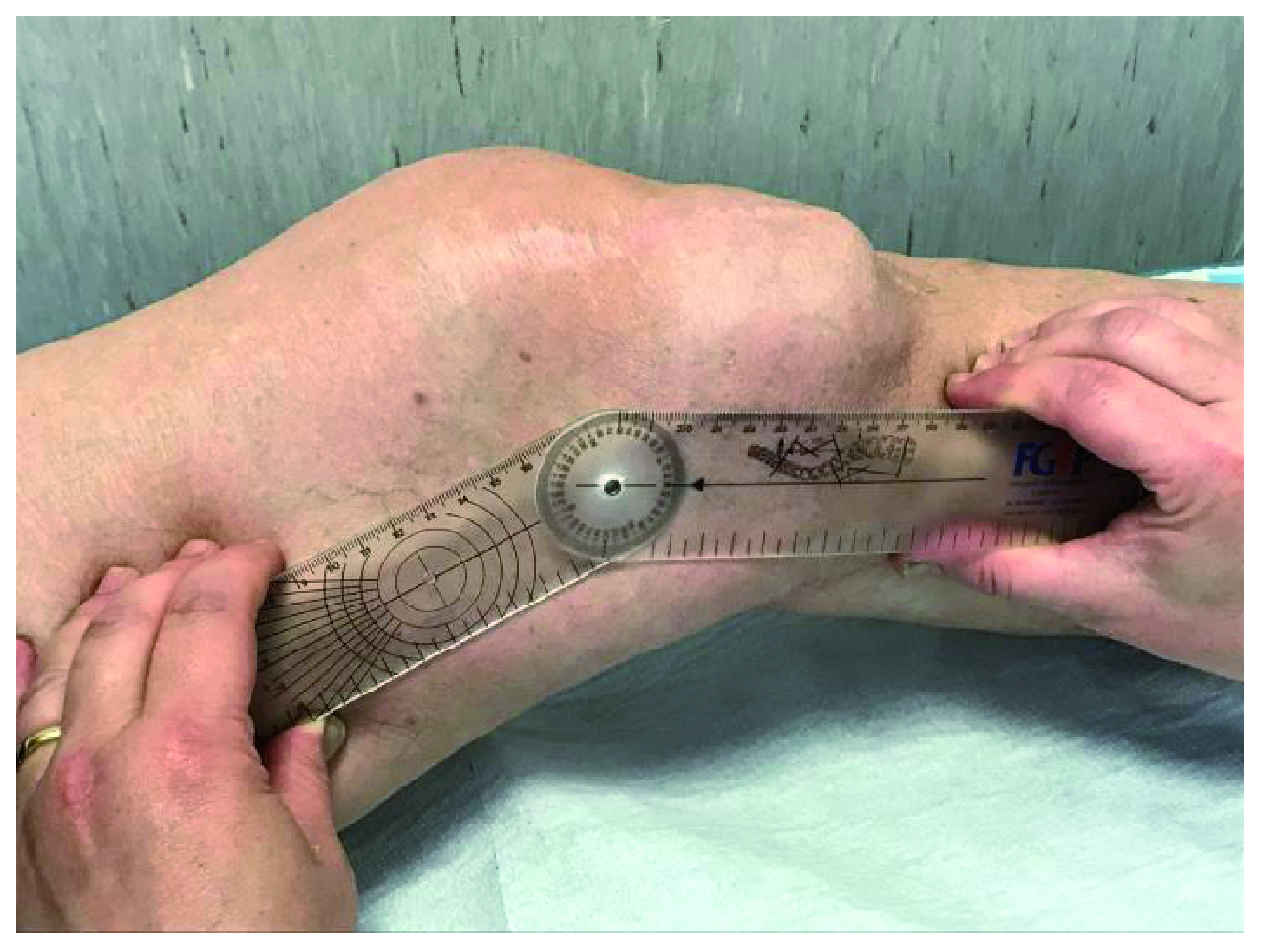
Malformations of the left lower arms, (hip, knee and ankle), evident at the joint and periarticular level, where diffused exostoses are present.

**Figure 4.**
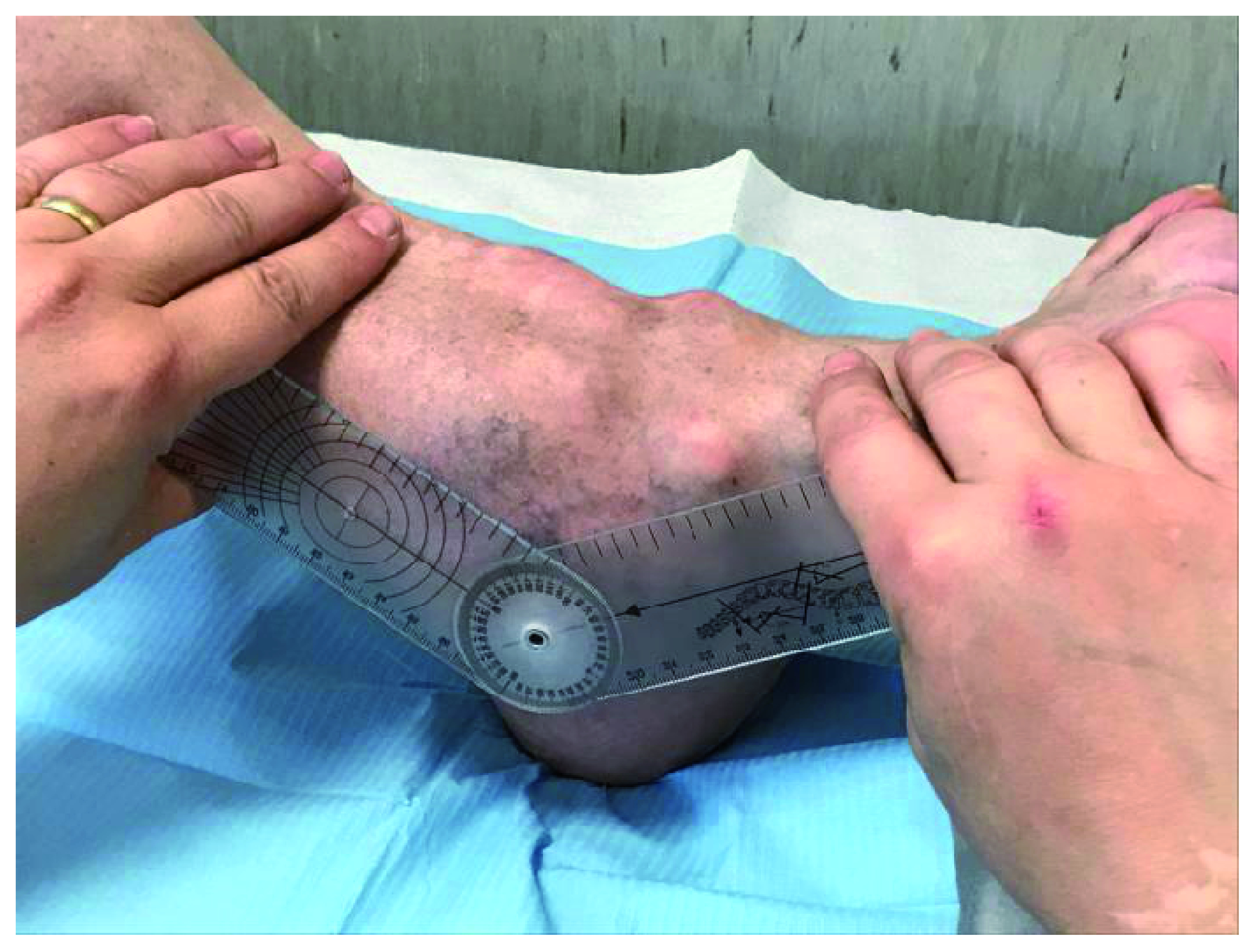
Complete ankylosis of the plantar flexion of 32 degrees.

In 1989 the tibial area was the site of surgical removal of an intra and extra capsular fibrolipomatous bulk, about 5 cm in diameter, excision of an intra-articular mobile body, medial transposition of the patellar tendon with insertion of metal plaque and transposition of the tendon insertion of the vast medial. The ankle condition appeared even more severe, with full ankylosis in plantar flexion of 32 degrees. Both the ankle and the foot appeared to be twice the size of the contralateral limb for bone hyperplasia and connective tissue ( Figure 3, Figure 4).

***Movement of the patient***. The patient has a gait abnormality (limp, asymmetrical, slower than normal), which currently does not require aids on level ground. Uphill movement causes bending of the torso with increased effort, therefore where the risk of falling increases (e.g. while descending the stairs) support with a stick is required.

The patient, due to the high risk of falling while using the stairs, has to be highly concentrated and a handrail is required. The deficit is increased by the sharp decrease in proprioceptive and exteroceptive sensitivity of the left foot plantar, which is housed in custom-made orthopedic footwear.

The impairment of walking leads to functional overload of the lower right limb with episodes of low back pain and homolateral coxalgia.

### Diagnostic assessment

Our patient reported that based on the clinical characteristics, a diagnosis of proteus syndrome was issued at 18 months.

However, a recent genetic study performed in the patient at the age of 54 years highlighted the absence of the characteristic mutation present in proteus syndrome, suggesting a different diagnosis. The detection of the PIK3CA gene mutation (c.3140A> G), together with the clinical aspects of the patient, suggests a diagnosis of fibro-adipose hyperplasia syndrome (FHS), characterized by a congenital, progressive and localized overgrowth of fibrous and adipose tissue and bone. The FHS is part of the broader category of syndromes termed PIK3CA-related overgrowth spectrum (PROS), which includes LOVES, FHS, macrodactyly and megalocephaly syndrome. To date, the only treatment expected to slow down uncontrolled tissue growth is surgical resection. The aim of genetic research is to use ‘target therapies’, such as molecular inhibitors of the PI3K/AKT /mTOR pathway, currently used in oncology, which may slow down if not reverse the characteristic hyper-growth of these syndromes. Objective assessment of neuromuscular function can help the occupational medicine specialist to better evaluate global impairment and the presence of symptoms ^5^.

### Workplace intervention

We performed no therapeutic intervention since no health issue was present at the clinical evaluation. However, since the patient’s job had recently changed from administrative tasks to an activity characterized by more frequent contact with the public (more standing up and moving around for the worker), we ergonomically evaluated and adjusted the patient’s workplace.

The patient’s work environment consists of an office on the second floor where the patient carries out activities on a PC connected to the administration of the library where he works. His work chair is comfortable, allows height adjustment and an inclination of the backrest; the desk is spacious, ergonomically organized and allows comfortable housing of the legs of the subject. To allow the comfortable support of the foot, a stool was provided to the patient.

Movements at the workplace are very limited and of short duration; however, given the limitations of the patient’s movement, the use of the stairs must be limited and the use of a dedicated elevator is required. Therefore, to allow travel to and from the workplace, the patient was reserved a flat parking space, and an elevator located at the parking level to his workstation was provided, as well as to the entire library area and related offices.

The main occupational concern that could create critical issues with patients with this type of syndrome with restricted movement would be in the event of an emergency exodus, e.g. fire. Therefore, various measures and simulations were put in place to assess and reduce the risk for this patient in an event such as this.

For our patient, the maximum distance between a safe place to go to in event of an evacuation (as determined by the fire department, which was on a level) and the patient’s workstation or other places of work the patient occasionally frequented, such as reading room and book store, was <30 meters. A simulation was carried out at the patient’s workplace by the patient to assess the time taken for arrival at the safe place from the usual workstation (30 seconds) and from a more distant location (45 seconds), which seem to be acceptable times for the worker to keep in safe. 

The presence of an automatic fire detection and extinguishing system may constitute a risk element for our patient as it maked the floors more slippery, increasing the risk of falls. Therefore, a training plan aimed at emergency evacuation and a specific exercise was planned for the entire staff. In addition, two work colleagues were trained specifically for the assistance of the patient, and a tested evacuation and transport chair (model PRO-SKID by SPENCER) was purchased to descend the external fire escape stairs.

### Follow-up and outcomes

We assessed the outcomes of the change in workstation at 6 months. The patient reported increased work comfort and a regular use of the elevator located at the parking level to reach his workstation. He reported no falls during the last 6 months and no accidents have occurred. 

## Discussion

Overgrowth syndrome is a clinical condition characterized by progressive and uncontrolled growth of different parts of the body. It can affect the skeleton, skin, subcutaneous tissue, and central nervous system. The onset is mostly between 6 and 18 months of life, with asymmetric overgrowth, usually of the hands and feet. Macrodactyly and hemi-hypertrophy of bone and connective tissue are the most common onset symptoms.

In 1998 the National Institutes of Health (Bethesda, MD, USA) defined guidelines to base the diagnosis of this syndrome on clinical criteria. In order for the diagnosis of proteus syndrome to be considered valid, the following must be present ( Table 1): all three general criteria plus a criterion relating to category A; two criteria of category B; three criteria of category C. The absence of such requirements can lead to the diagnosis of proteus-like syndrome. In the case of our patient, the clinical diagnosis was carried out in accordance with the general criteria, with the specific criteria of class A and a criterion of class B. For the diagnostic confirmation it is necessary to identify the mutation of the gene responsible for the syndrome. Proteus syndrome is an extremely rare and complex disease (1/1,000,000 newborns) and currently only 120 cases in the world are recognized ^2^.

**Table 1.** Diagnostic criteria of proteus syndrome.

*General criteria:* • Mosaic distribution of lesions; • Sporadic recurrence; • Progressive course.
*Criteria for evolutionary framework:* * • Category A:* requires, as the only criterion, the presence of cerebriform nevi of connective tissue • *Category B:* requires the presence of disproportionate and asymmetric overgrowth, or linear epidermal nevus, or specific tumors before the 2nd decade, bilateral cystadenoma of the ovaries or monomorphic adenoma of the parathyroid glands. • *Category C:* requires the presence of vascular malformations, adipose dysregulation, typical facial phenotype

Recent studies have shown that proteus syndrome is caused by a mutation of the pathway of the phosphatidylinositol-3-kinase signal (PI3K)-AKT ^6^. Patients are affected by somatic activating mutation of the oncogene AKT1, present in the form of mosaicism. AKT (also known as protein-kinase B or PKB) is a cytosolic protein that plays a key role in the PI3K\Akt pathway. Its activity consists of the phosphorylation of various protein substrates in the serine and threonine residues, often leading to their inactivation. The activation of this oncogene triggers biochemical pathways that lead to cell proliferation and resistance to apoptosis.

Treatment of overgrowth syndrome requires a multidisciplinary approach. Interventions for the control of overgrowth are epiphysiodesis and amputation in severe cases. Physiotherapy and occupational therapy are very important to avoid joint ankylosis and to maintain an adequate self-functional autonomy. Orthoses and custom-made shoes may be needed.

For CCTNs, which are a typical manifestation of this syndrome, dermatological treatment is required; the lesions must be removed surgically in the case of suspected malignancy or if the deformities and/or pain are significant. In the case of deep venous thrombosis and pulmonary embolism, guidelines for anticoagulant therapy should be applied promptly. Regular screening tests must be performed due to a predisposition to neoplasms.

Patients and their families can benefit from psychosocial counseling. Annual physical and radiological examination is recommended. Given the consequent impossibility of placing the patients in heavy manual activities, the employment of these patients is limited to office activities. In cases of overgrowth syndromes, the exact identification of the limitations presented by the patient and observations about ambulatory functions must be carefully evaluated in order to modulate the work environment.

## Ethical approval

The occupational therapy protocol was approved by the local ethical committee of Tor Vergata Policlinic and written informed consent was obtained by the patient for their participation in the study.

## Consent

Written informed consent for publication of their clinical details and/or clinical images was obtained from the patient.

## Data availability

All data underlying the results are available as part of the article and no additional source data are required.
