# Supplementary material for: Case Report: Occupational therapy in a patient with an overgrowth syndrome that restricts movement
Source: F1000Res. 2021 Aug 23;8:2050. Originally published 2019 Dec 3. [Version 2] doi: 10.12688/f1000research.21348.2 (PMC8329603; doi:10.12688/f1000research.21348.2)
Supplement: Peer review history

# Peer review: Reviewer response for version 1

**DOI:** 10.5256/f1000research.23509.r90062

**Published:** 2021-08-02

**Authors:** Enrico Maria Staderini

**Affiliations:** 1 Western Switzerland University of Applied Sciences-Haute Ecole d'Ingénierie et de Gestion du Canton de Vaud (HEIG-VD), Yverdon-les-Bains, Switzerland; 2 Healthy World Association - Switzerland, Yverdon-les-Bains, Switzerland

Nice report of a quite rare case of overgrowth syndrome quite severely involving lower limbs. Very good description of the case and scientific framing of the syndrome. The description of the original workplace intervention may be of interest to other readers.

 A few points to modify:

1. In various parts of the text "lower left arm" is indicated while it should be much more appropriated to write "lower left leg" or "lower left limb" instead (please fix this point).
2. In the abstract the age of the patient is given as 57 years old, while in the text (first line Case report Patient information paragraph) the age of the same patient is given a 59 years old (please fix this point).

Are enough details provided of any physical examination and diagnostic tests, treatment given and outcomes?

Yes

Is the case presented with sufficient detail to be useful for other practitioners?

Yes

Is sufficient discussion included of the importance of the findings and their relevance to future understanding of disease processes, diagnosis or treatment?

Yes

Is the background of the case’s history and progression described in sufficient detail?

Yes

Reviewer Expertise:

Biomedical technology, medicine, cardiology

I confirm that I have read this submission and believe that I have an appropriate level of expertise to confirm that it is of an acceptable scientific standard.

# Response

**Published:** 2021-08-02

**Authors:** luca coppeta

**Affiliations:** tor vergata university, Italy

Thank you for your suggestions. We have provided to corrections the text.

# Peer review: Reviewer response for version 1

**DOI:** 10.5256/f1000research.23509.r57442

**Published:** 2019-12-04

**Authors:** Nicola Magnavita

**Affiliations:** 1 Post-graduate School of Occupational Health, Università Cattolica del Sacro Cuore, Rome, Italy; 2 Fondazione Policlinico Gemelli IRCCS Roma, Rome, Italy

The article is interesting because, beyond the description of the case which is infrequent in itself, it offers interesting hints about ergonomic interventions that can be useful to improve the employment conditions of those suffering from this disease.

 Since the pathology is progressive and the worker will also undergo periodic checks at the workplace in the future, the authors could say whether, from a total worker health perspective, they have planned interventions aimed at increasing physical activity, avoiding weight accumulation, improve joint mobilisation.

Are enough details provided of any physical examination and diagnostic tests, treatment given and outcomes?

Yes

Is the case presented with sufficient detail to be useful for other practitioners?

Yes

Is sufficient discussion included of the importance of the findings and their relevance to future understanding of disease processes, diagnosis or treatment?

Yes

Is the background of the case’s history and progression described in sufficient detail?

Yes

Reviewer Expertise:

occupational health

I confirm that I have read this submission and believe that I have an appropriate level of expertise to confirm that it is of an acceptable scientific standard.
